# Development and validation of the BPDKC model for predicting sarcopenic knee osteoarthritis in community-dwelling older adults: a cross-sectional study

**DOI:** 10.1186/s12891-025-09311-6

**Published:** 2025-11-26

**Authors:** Guang-Ming Liu, Bo Sun, Jia-Yu Yang, Cheng-Yi Rong, Ying Wang, Yu-Min Hu, Lin-Fei Yang, Jian Pang, Hong-Sheng Zhan

**Affiliations:** 1 Shuguang Hospital Affiliated to Shanghai University of Traditional Chinese Medicine, Shanghai, 201203 China; 2 Xiangshan Hospital of Traditional Chinese Medicine, Huangpu District, Shanghai, 201203 China; 3 Yuyuan Community Health Service Center, Huangpu District, Shanghai, 200010 China

## Abstract

**Background:**

Sarcopenia and knee osteoarthritis frequently coexist as sarcopenic knee osteoarthritis (SPKOA), a clinically significant and burdensome condition among older adults. Despite its high prevalence and functional impact, no validated, practical tool exists for early identification of SPKOA in community settings. This study aimed to develop and internally validate a parsimonious, clinically applicable prediction model for SPKOA risk stratification in community-dwelling older adults.

**Methods:**

We conducted a cross-sectional analysis of 640 community-dwelling adults aged 65–85 years, recruited between March and October 2024 from the Yuyuan Community Health Service Center in Shanghai, China (median age: 71 years; 57% female). SPKOA was diagnosed using established criteria for both sarcopenia and knee osteoarthritis. Participants were randomly split (7:3) into training (*n* = 448) and internal test (*n* = 192) sets. In the training set, we employed a hybrid variable selection strategy: age, gender, BMI, physical activity (PA), diabetes mellitus (DM), Knee Visual Analog Scale(KVAS), and calf circumference (CC) were prespecified based on clinical relevance; remaining candidate variables underwent univariate screening (*P* < 0.05), followed by forward stepwise logistic regression (entry: *P* < 0.05; removal: *P* > 0.10). Three candidate models were derived and compared: Model 1 (7 predictors: CC, KVAS, BMI, DM, PA, osteoporosis, age); Model 2 — the BPDKC model (5 predictors: BMI, PA, DM, KVAS, CC); and Model 3 (5 predictors: DM, PA, KVAS, CC, age). Model performance was evaluated using discrimination (AUC with 95% CI), calibration (Brier score, calibration plots), clinical utility (decision curve analysis). The optimal model was translated into a nomogram and an interactive web-based calculator.

**Results:**

Among 640 participants, 12.5% (*n* = 80) met SPKOA criteria, with balanced prevalence across training (12.5%, *n* = 56) and test sets (12.5%, *n* = 24). In the training set, all models showed strong discrimination (Model 1 AUC = 0.953; Model 2 AUC = 0.951; Model 3 AUC = 0.934) and excellent calibration (Brier scores: 0.053–0.062). However, in the internal test set, Model 2 (BPDKC) demonstrated the best generalizability and balance of performance: AUC = 0.861 (95% CI: 0.795–0.926), specificity = 94.6% (95% CI: 90.1–97.5%), precision = 50.0% (95% CI: 26.0–74.0%), F1-score = 0.429 (95% CI: 0.243–0.618), and Brier score = 0.1038 (95% CI: 0.068–0.139). Although sensitivity remained modest (37.5%, 95% CI: 18.8–59.4%), calibration curves and decision curve analysis confirmed robust risk estimation and superior net clinical benefit across clinically relevant risk thresholds (10–40%). Model 2 also achieved the best trade-off between performance and parsimony (AIC = 178.41 vs. Model 3 AIC = 194.79). A user-friendly web calculator implementing the BPDKC model is freely accessible at (https://lgmyyyy.shinyapps.io/bpdkc_spkoa_app/) .

**Conclusion:**

The BPDKC model is a simple, internally validated, and clinically practical tool for SPKOA risk prediction in community-dwelling older adults. Its high specificity and calibration support its utility in ruling out low-risk individuals, while its web-based implementation facilitates integration into primary care workflows. Although sensitivity remains limited—reflecting the challenge of case-finding in low-prevalence settings—the model offers meaningful clinical value for targeted screening and early intervention. External validation in diverse populations is recommended prior to broader implementation.

**Supplementary Information:**

The online version contains supplementary material available at 10.1186/s12891-025-09311-6.

## Introduction

Sarcopenia (SP; ICD-11: M62.84) and knee osteoarthritis (KOA; ICD-11: M17.9) are two highly prevalent geriatric syndromes that significantly impair mobility, increase fall risk, and reduce quality of life in older adults. The global burden of these conditions is rising rapidly, driven by population aging and sedentary lifestyles.

SP is characterized by a progressive and generalized loss of skeletal muscle mass, strength, and physical performanceis characterized by persistent, systemic loss of skeletal muscle mass, strength, and physical performance [1, 2]. The European Working Group on Sarcopenia in Older People 2 (EWGSOP2) has established widely aCCepted diagnostic criteria, which emphasize low muscle strength as the primary indicator, confirmed by low muscle quantity or quality (e.g., measured by appendicular skeletal muscle mass index, ASMI) [3]. Management typically involves resistance training, protein supplementation, and multimodal interventions targeting muscle function and metabolic health.

KOA is a bone and joint disease characterized by the degradation of articular cartilage. Patients with KOA often complain of joint pain, stiffness, and difficulty with purposeful movement [4]. Its pathological features primarily involve articular cartilage degeneration, accompanied by secondary changes such as osteophyte formation, subchondral bone sclerosis, and joint inflammation. Management of KOA includes non-surgical and surgical approaches [5, 6]. Non-surgical treatment encompasses patient education, lifestyle modification, physical therapy, orthotic devices, and nonsteroidal anti-inflammatory drugs (NSAIDs), and is recommended as first-line therapy [7]. While surgical intervention is reserved for advanced or refractory cases [8], Infiltrative therapies have emerged as effective second-line options [9, 10]. Additionally, novel therapeutic approaches—including sprifermin, bone morphogenetic protein-7 (BMP-7), monoclonal antibodies, and gene therapy—are under investigation [11].

The coexistence of SP and KOA, commonly referred to as sarcopenic knee osteoarthritis (SPKOA), has gained increasing attention in recent years [12]. Emerging evidence indicates a complex interplay between these two conditions, with a potential bidirectional relationship. On one hand, the altered biomechanics and reduced physical activity associated with KOA may contribute to the development and progression of SP [13, 14]. Chronic knee pain and joint instability can limit mobility, leading to disuse atrophy of the surrounding musculature, particularly the quadriceps [15]. On the other hand, sarcopenia-related muscle weakness and impaired joint stability may increase mechanical stress on the knee joint, thereby accelerating cartilage degeneration and worsening osteoarthritis [16].

Studies have reported that the prevalence of SPKOA ranges from 28% to over 60% among individuals with knee osteoarthritis [17, 18]. Population-based estimates in community-dwelling older adults suggest a comorbidity rate of approximately 8.6% [19], highlighting its substantial public health impact.

However, the underlying mechanisms linking SP and KOA remain incompletely understood, and there is currently no consensus on optimal diagnostic criteria or management strategies for SPKOA [20].

Clinical prediction models (CPMs) have become increasingly important tools for risk stratification and personalized healthcare across a wide range of medical conditions. By integrating readily available clinical variables, CPMs enable early identification of high-risk individuals, support clinical decision-making, and facilitate targeted interventions—particularly in primary care and community settings [21]. Commonly used clinical tools for sarcopenia screening in community settings include calf circumference (CC). However, these tools have limitations in sensitivity, specificity, and ability to quantify risk gradients. Recently, studies have shown that simple anthropometric variables—such as body weight, mid-upper arm circumference, and additional limb measurements beyond CC—can be integrated into machine learning algorithms to improve the accuracy of SP diagnosis [22–25].

In the context of SPKOA, the development of a reliable clinical prediction model (CPM) could facilitate the early identification of high-risk individuals, enabling timely interventions and potentially slowing disease progression. However, existing CPMs for SP or KOA typically address these conditions in isolation, and there is a notable lack of models specifically designed to capture the complex, interrelated phenotype of SPKOA [26, 27].

Therefore, this study aims to estimate the prevalence of SPKOA in a Chinese community-dwelling older population, identify its independent risk factors, and develop and validate a simple, clinically applicable prediction model—the BPDKC model (based on body mass index (BMI), Physical activity (PA), DM Mellitus (DM), Knee Visual Analogue Scale (KVAS), and CC)—which is further translated into user-friendly tools, including a nomogram and a web-based calculator, to support real-world implementation.

## Data and methods

### Study design

This cross-sectional study was retrospectively registered in the Chinese Clinical Trial Registry (ChiCTR; registration number: ChiCTR2500108158) after study completion on August 26, 2025 (available at: https://www.chictr.org.cn/showproj.aspx?proj=214137). Data were collected between March and October 2024. The study protocol was approved by the Ethics Committee of Xiangshan Hospital of Traditional Chinese Medicine (Approval No.: XSEC2023010). Written informed consent was obtained from all participants prior to enrollment. All data were anonymized and handled in strict compliance with national data protection regulations; no identifiable personal information was collected or stored.

### Study population

#### Participants

**Fig. 1:**
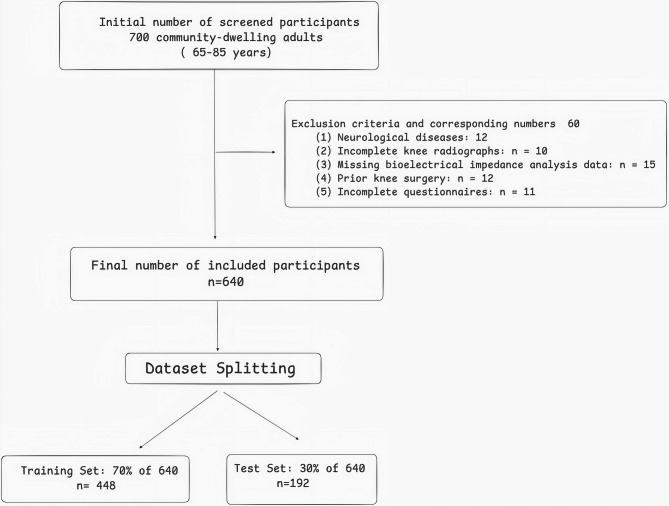
Initial screening and dataset splitting

This cross-sectional study was conducted at the Yuyuan Community Health Service Center in Shanghai, China, from March to October 2024, in accordance with the Strengthening the Reporting of Observational Studies in Epidemiology (STROBE) guidelines [28]. A total of 700 community-dwelling older adults aged 65–85 years were initially screened. Of these, 60 were excluded due to neurological disorders (*n* = 12), prior knee surgery (*n* = 8), incomplete knee radiographs (*n* = 15), missing bioelectrical impedance analysis (BIA) data (*n* = 18), or incomplete questionnaires (*n* = 7). The final analytical sample comprised 640 participants. The sample was randomly allocated to a training set (70%, *n* = 448) and an internal test set (30%, *n* = 192). Participant flow is illustrated in Fig. 1.

#### Diagnostic criteria

SP was defined using a combination of the revised EWGSOP2 [3] and AWGS 2019 criteria [1], requiring low appendicular skeletal muscle index (ASMI; measured by bioelectrical impedance analysis: <7.0 kg/m² in men, < 5.5 kg/m² in women) plus either low grip strength (< 28 kg in men, < 18 kg in women) or slow gait speed (< 1.0 m/s). KOA was diagnosed according to the American College of Rheumatology (ACR clinical and radiographic criteria 1995) criteria [29], defined as knee pain in the past month plus at least one of the following: age ≥ 50 years, morning stiffness ≤ 30 min, crepitus, or or radiographic evidence of osteoarthritis (joint space narrowing, subchondral sclerosis, cyst formation, or osteophytes) on weight-bearing anteroposterior knee radiographs. Radiographs were independently reviewed by two musculoskeletal radiologists blinded to clinical data; a Kellgren–Lawrence (KL) grade ≥ 2 was considered positive for radiographic OA. SPKOA was diagnosed when both SP and KOA were present. All diagnoses were made independently by two orthopedic specialists with 12–15 years of experience, with excellent inter-rater reliability.

#### Inclusion criteria

Participants were included if they: (1) were aged 65–85 years; (2) could independently complete all study assessments, including the 6-meter walk test and questionnaires; and (3) provided written informed consent.

#### Exclusion criteria

Participants were excluded if they had any of the following: (1) Neurological or musculoskeletal conditions that could confound SP or KOA assessment: cognitive impairment (Mini-Mental State Examination score < 24, adjusted for education level), history of stroke, Parkinson’s disease, balance disorders, or metal implants; (2) Severe functional dependency: requiring full assistance for basic activities of daily living (ADLs); (3) Prior knee surgery; (4) Incomplete or missing data: non-diagnostic knee radiographs, missing BIA data, or questionnaires with > 10% of core items unanswered.

### Variable assessment

A total of 17 candidate variables were assessed during a single study visit (≤ 2 h) to minimize intra-individual variability. Demographic characteristics (age, sex, BMI) were collected via face-to-face interviews. Comorbidities—including DM, hypertension, coronary heart disease, and osteoporosis—were verified against electronic medical records.

Lifestyle factors comprised: (1) physical activity (PA), assessed using the WHO Global Physical Activity Questionnaire (GPAQ) and categorized as low (< 600 MET-min/week) or high (≥ 600 MET-min/week); (2) smoking and alcohol consumption (current use: yes/no); and (3) dietary pattern, self-reported as “balanced” (adequate protein and vegetable intake) or “imbalanced.”

Knee-specific outcomes included: (1) pain intensity, measured using the Knee Visual Analog Scale (KVAS; 0 = no pain, 10 = worst imaginable pain); and (2) radiographic severity, graded using the KL system [30] on weight-bearing bilateral knee radiographs and grouped as mild (KL 1–2), moderate (KL 3), or severe (KL 4).

Anthropometric and muscle-related measures were: (1) CC, measured at the maximal girth of the more symptomatic leg (or right leg if symptoms were bilateral and equal) using a non-elastic tape; (2) grip strength, recorded as the highest of three trials using a digital dynamometer; (3) gait speed, assessed over a 6-meter walk at usual pace; and (4) ASMI, derived from BIA. All continuous variables were summarized as median (Q1-Q3).

### Statistical analysis

All statistical analyses were conducted using R software (version 4.4.2; R Foundation for Statistical Computing, Vienna, Austria), with key packages including caret (data partitioning), rms (logistic regression modeling with restricted cubic splines and nomogram development), pROC (ROC analysis), and gtsummary (standardized reporting). A fixed random seed (123) was set to ensure reproducibility.

Baseline characteristics between SPKOA and non-SPKOA participants were compared using Chi-squared tests for categorical variables and Wilcoxon rank-sum tests for continuous variables. Continuous predictors were modeled flexibly using restricted cubic splines (RCS) with 5 knots; nonlinearity was tested via likelihood ratio test comparing RCS and linear models.

The full dataset was randomly split into a training set (70%) for model development and a test set (30%) reserved exclusively for internal validation. The training set contained 80 SPKOA cases, yielding an events-per-variable (EPV) ratio of ~ 10:1 for up to 8 predictors, which was considered sufficient for stable estimation [31].

Model performance was evaluated in the test set across three domains: (1) discrimination, using the area under the receiver operating characteristic curve (AUC) with 95% confidence interval; (2) calibration, assessed via calibration plot and calibration slope; and (3) clinical utility, evaluated using Decision Curve Analysis (DCA) across threshold probabilities from 5% to 90%. Classification metrics (sensitivity, specificity, positive/negative predictive values, F1-score) were derived from a confusion matrix using the Youden index–optimal probability threshold determined in the training set.

A nomogram was constructed for clinical visualization. Additionally, an interactive web-based risk calculator was developed using R Shiny.

## Results

### Study population characteristics

A total of 640 community-dwelling older Chinese adults were included, with a median age of 71.0 years (Q1–Q3: 68.0–74.0) and 57.7% female. The prevalence of SPKOA was 12.5% (80/640). Participants were randomly allocated to a training set (*n* = 448, 70%) and a test set (*n* = 192, 30%). Most baseline characteristics were balanced between the two sets (all *p* > 0.05), except for gender, which showed a statistically significant difference (*p* = 0.033) (Supplementary table 1). and Baseline Characteristics and Univariate/Multivariate Logistic Regression Analyses for SPKOA in the train set (Supplementary table 2).

In univariate analysis across the total set, 12 variables showed association with SPKOA (*p* < 0.10). After multivariate adjustment, nine variables remained independently associated with SPKOA (*p* < 0.05): Gender(female) showed a counterintuitively low adjusted OR (aOR = 0.05, 95% CI: 0.01–0.20), likely due to multicollinearity with body composition measures (e.g., ASMI, calf circumference) that were simultaneously included in the model — a known statistical artifact when highly correlated predictors compete for variance explanation. Other significant factors included high physical activity (aOR = 0.35, 0.15–0.80), DM (aOR = 4.84, 2.15–11.50), coronary heart disease (aOR = 0.37, 0.13–0.95), older age (aOR = 1.10/year, 1.01–1.21), higher KVAS (aOR = 1.87, 1.49–2.41), lower paces per minute (aOR = 0.00, 0.00–0.04), lower ASMI (aOR = 0.04, 0.01–0.10), and smaller calf circumference (aOR = 0.64, 0.53–0.76). Grip strength lost significance after adjustment, while coronary heart disease emerged only in multivariate modeling, suggesting confounding in unadjusted estimates.

### Variable selection strategy and final predictor set

We implemented a targeted variable selection process for the SPKOA prediction model, using only the training set (*n* = 448) to avoid overfitting (Supplementary table 3). First, 17 candidate variables identified through univariate screening were subjected to full logistic regression. Guided by the criterion of multivariate regression *p* < 0.05, 8 variables were retained: Gender, Diet, DM, CHD, KVAS, Paces, ASMI, and CC. Thirteen variables were excluded for failing to meet this threshold.

Paces and ASMI were then removed to eliminate circularity, as they are core diagnostic components of SP. undermining the model’s causal plausibility. Next, age, BMI, and physical activity were re-included based on strong epidemiological and biological evidence (all *p* < 0.001 in univariate analysis) and their established role in SPKOA pathogenesis; osteoporosis was also added due to its clinical relevance to both SP and KOA, expanding the candidate pool to 10 variables (Gender, Diet, DM, CHD, KVAS, CC BMI, Age, PA, Osteoporosis).

These 10 variables were entered into forward stepwise logistic regression (binomial glm; entry criterion: *p* < 0.05; removal criterion: *p* > 0.10) optimized by AIC minimization. Seven variables met the inclusion criterion and were retained as independent predictors (Supplementary table 4): CC (β=−0.687, *p* = 9.98 × 10⁻¹¹), KVAS (β = 0.802, *p* = 1.05 × 10⁻⁸), BMI (β=−0.280, *p* = 3.66 × 10⁻⁴), DM (Yes vs. No; β = 1.184, *p* = 0.006), physical activity (High vs. Low; β=−1.185, *p* = 0.010), osteoporosis (Yes vs. No; β = 1.120, *p* = 0.012), and age (β = 0.106, *p* = 0.029). Gender, diet, and coronary heart disease were excluded for not meeting the entry criterion.

The final model exhibited good fit (AIC = 172.55; residual deviance = 156.55, 440 degrees of freedom; null deviance = 337.59, 447 degrees of freedom) after 7 Fisher Scoring iterations. The final predictor set thus includes seven variables: CC, KVAS, BMI, DM, Physical Activity, Osteoporosis, and Age.

### Model comparison

Three logistic regression models were developed to predict SPKOA, with performance evaluated across training (*n* = 448) and test (*n* = 192) sets.

#### Model definitions

- Model 1: Full stepwise-derived model (7 predictors: CC, KVAS, BMI, DM, physical activity, osteoporosis, age).
- Model 2 (BPDKC Model): Simplified model (5 predictors: BMI, physical activity, DM, KVAS, CC).
- Model 3: age related model (5 predictors: DM, physical activity, KVAS, CC, age).

#### Performance summary

In the training set, all models showed excellent discrimination (AUC ≥ 0.934) and calibration. However, Model 1 exhibited marked overfitting, with AUC dropping from 0.953 to 0.835 in the test set. Model 2 demonstrated the best generalizability, achieving the highest test-set AUC (0.861, 95% CI: 0.795–0.926), high specificity (94.6%), and acceptable calibration (Brier score = 0.104). Model 3 performed worst, underscoring the importance of BMI.

Given its superior external validity, clinical simplicity, and avoidance of specialized diagnostics, Model 2 (BPDKC) was selected as the final prediction model. The acronym “BPDKC” denotes its five components: BMI, PA, DM, KVAS, and CC.

### Confusion matrix analysis

**Fig. 2:**
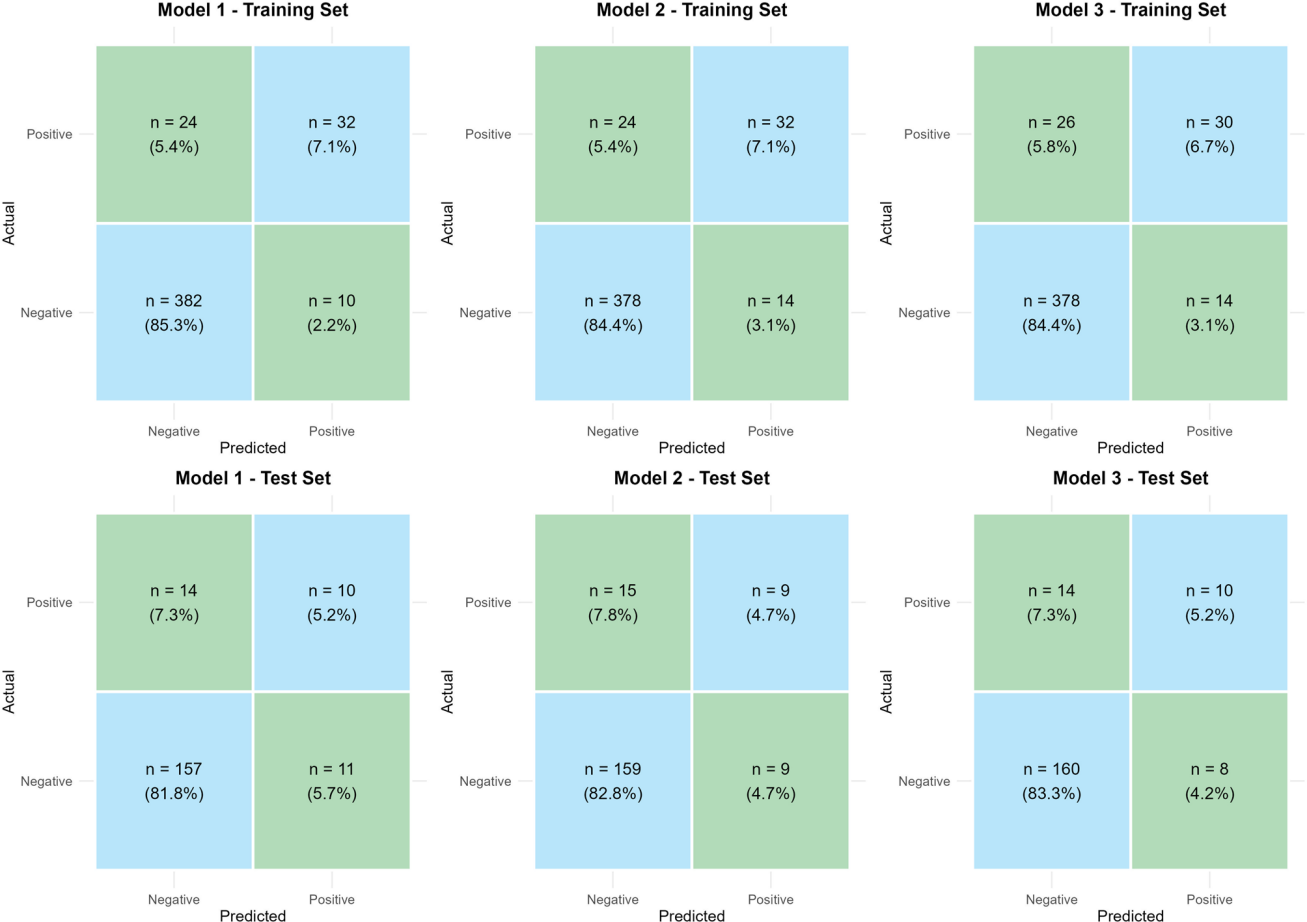
Confusion matrices for Model 1, Model 2, and Model 3 on training and test sets. Top row: Training set performance; Bottom row: Test set performance. True Positives (TP), False Positives (FP), False Negatives (FN), and True Negatives (TN) are shown as counts with row-wise percentages in parentheses. All models exhibit high specificity but suffer from substantial false negatives (> 80%), indicating limited sensitivity for detecting SPKOA cases despite strong negative-class identification

All models exhibited high specificity (training: 94.6–97.4%; test: 93.5–95.2%) but low sensitivity (training: 53.6–57.1%; test: 37.5–41.7%) (Fig. 2). False negative rates exceeded 80% in the test set, limiting utility for case finding despite strong non-SPKOA identification.

Model 3 showed marginal precision improvements (test: 55.6%), but no model achieved sufficient sensitivity for reliable screening. This reflects class imbalance (SPKOA prevalence = 12.5%) and highlights the need for future optimization.

### Model performance validation

#### Discriminative ability

**Fig. 3:**
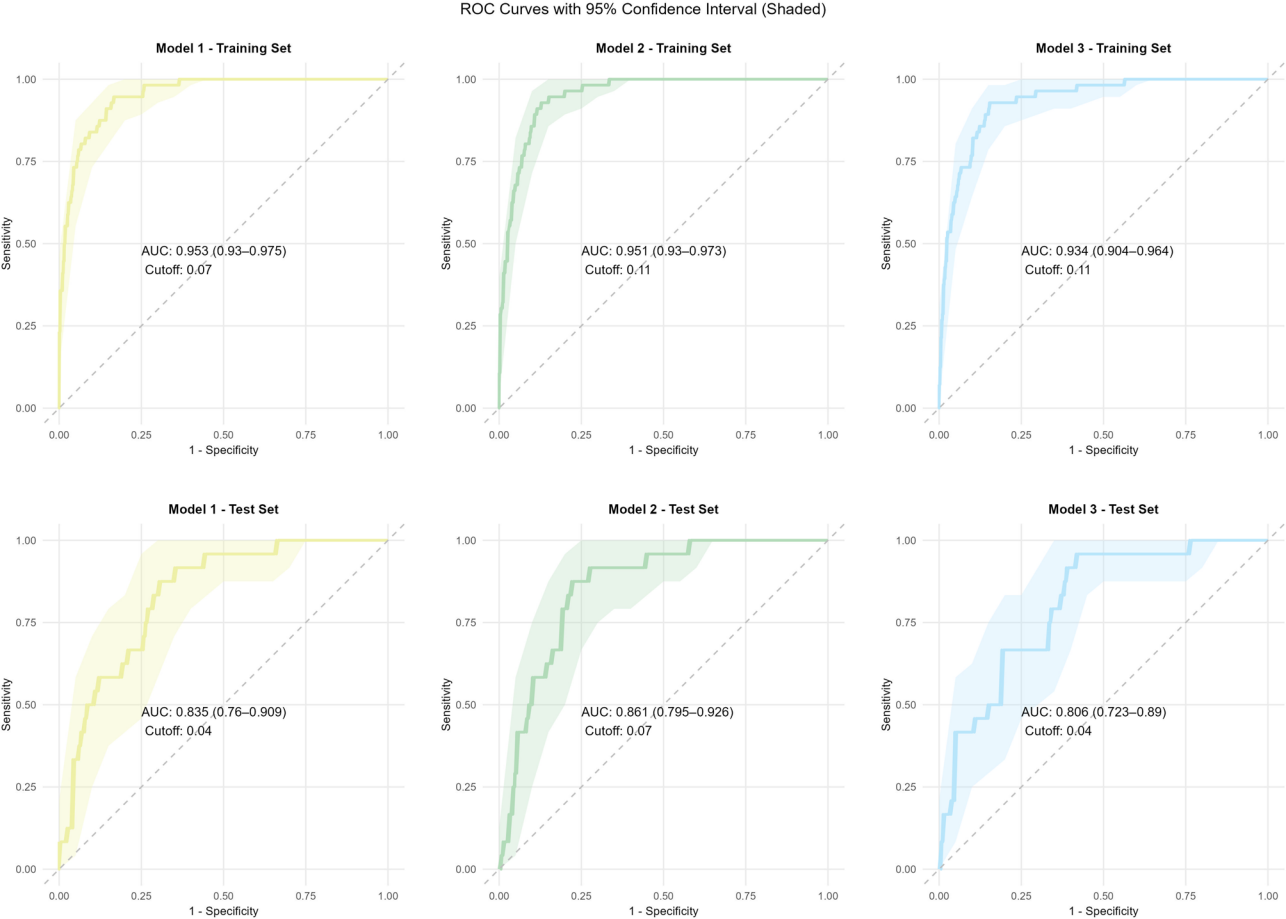
ROC curves of the 3 model. Our alternative models in the training vs. test set

Model 2 demonstrated excellent discrimination in the training set (AUC = 0.951, 95% CI: 0.951–0.973) and robust performance in the test set (AUC = 0.861, 95% CI: 0.861–0.926) (Fig. 3). At optimal cutoff, training-set sensitivity/specificity were 0.571/0.964, with an F1 score of 0.627 (95% CI: 0.506–0.729); test-set values were 0.375/0.946 and F1 = 0.429 (95% CI: 0.240–0.599).

#### Calibration

**Fig. 4:**
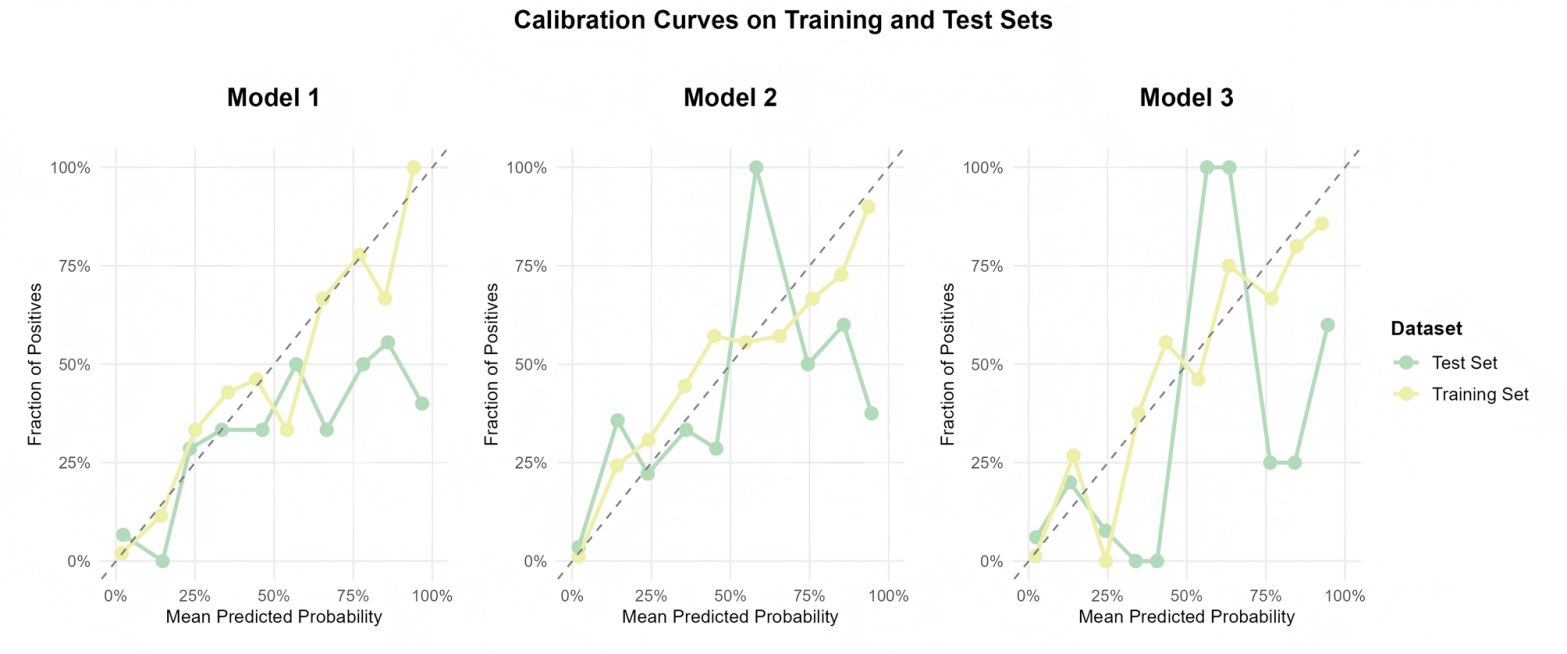
Calibration curves of the model. Calibration curves for Model 1, Model 2 (BPDKC), and Model 3 are depicted for both training and test datasets. The dashed line represents perfect calibration, where the mean predicted probability equals the observed fraction of positives. The solid lines represent the actual calibration curves for each model

Calibration curves showed strong alignment between predicted and observed SPKOA risks (Fig. 4). Model 2 had low error in the training set (MAE = 0.061, MSE = 0.0058) and test set (MAE = 0.063, MSE = 0.0061), confirming reliable risk estimation.

#### Clinical utility

**Fig. 5:**
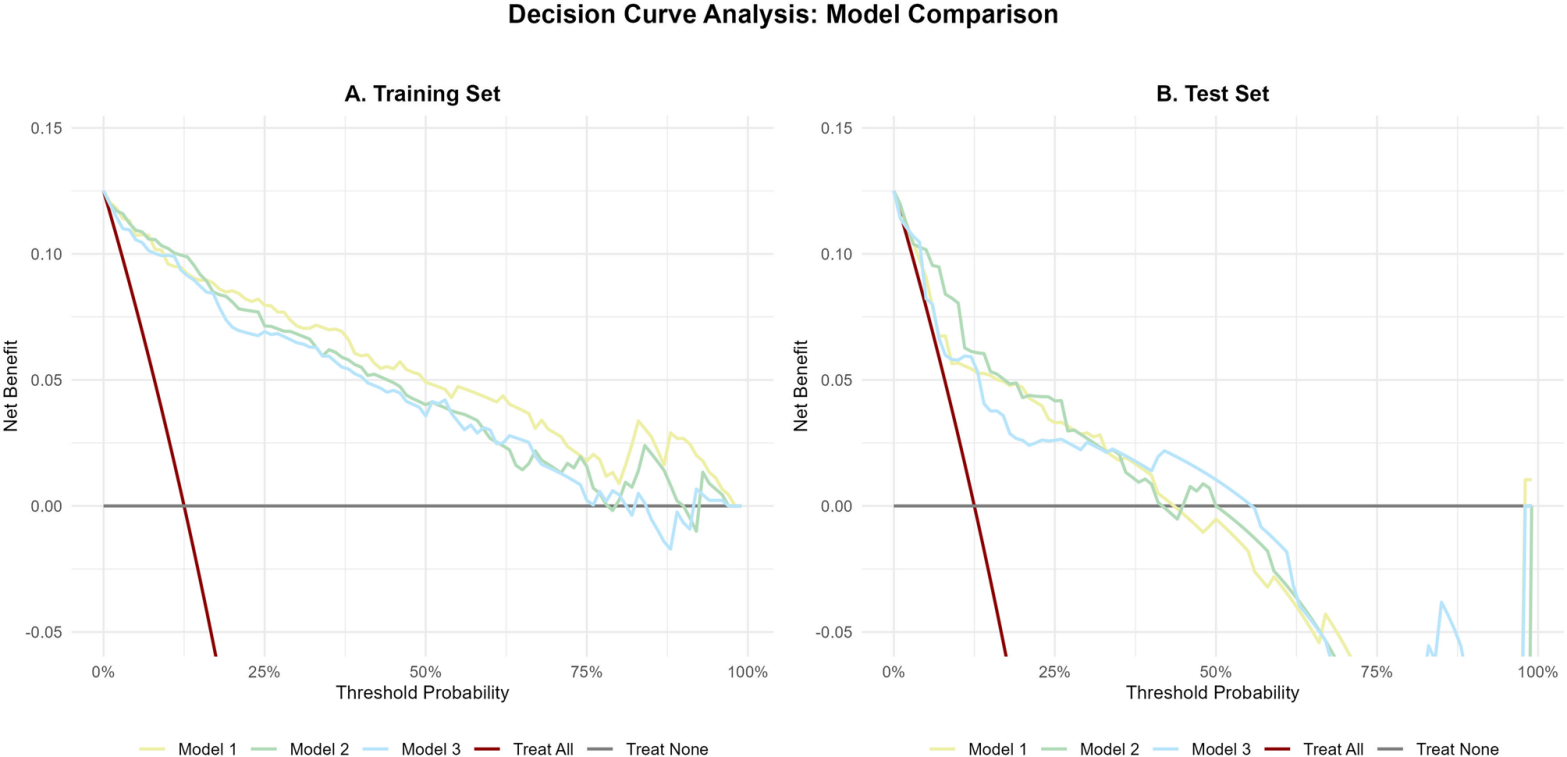
Decision Curve Analysis (DCA) Comparison. Decision curves for Model 1, Model 2 (BPDKC), and Model 3 are depicted for the training set (**A**) and test set (**B**). The net benefit is plotted against the threshold probability, with the “treat all” and “treat none” strategies serving as reference lines

Decision curve analysis (DCA) showed Model 2 had the highest net benefit across most threshold probabilities in both sets (Fig. 5). In the test set, it outperformed Models 1 and 3, particularly at 10–40% thresholds (relevant for community screening), supporting its clinical value.

#### Nonlinear predictor associations

**Fig. 6:**
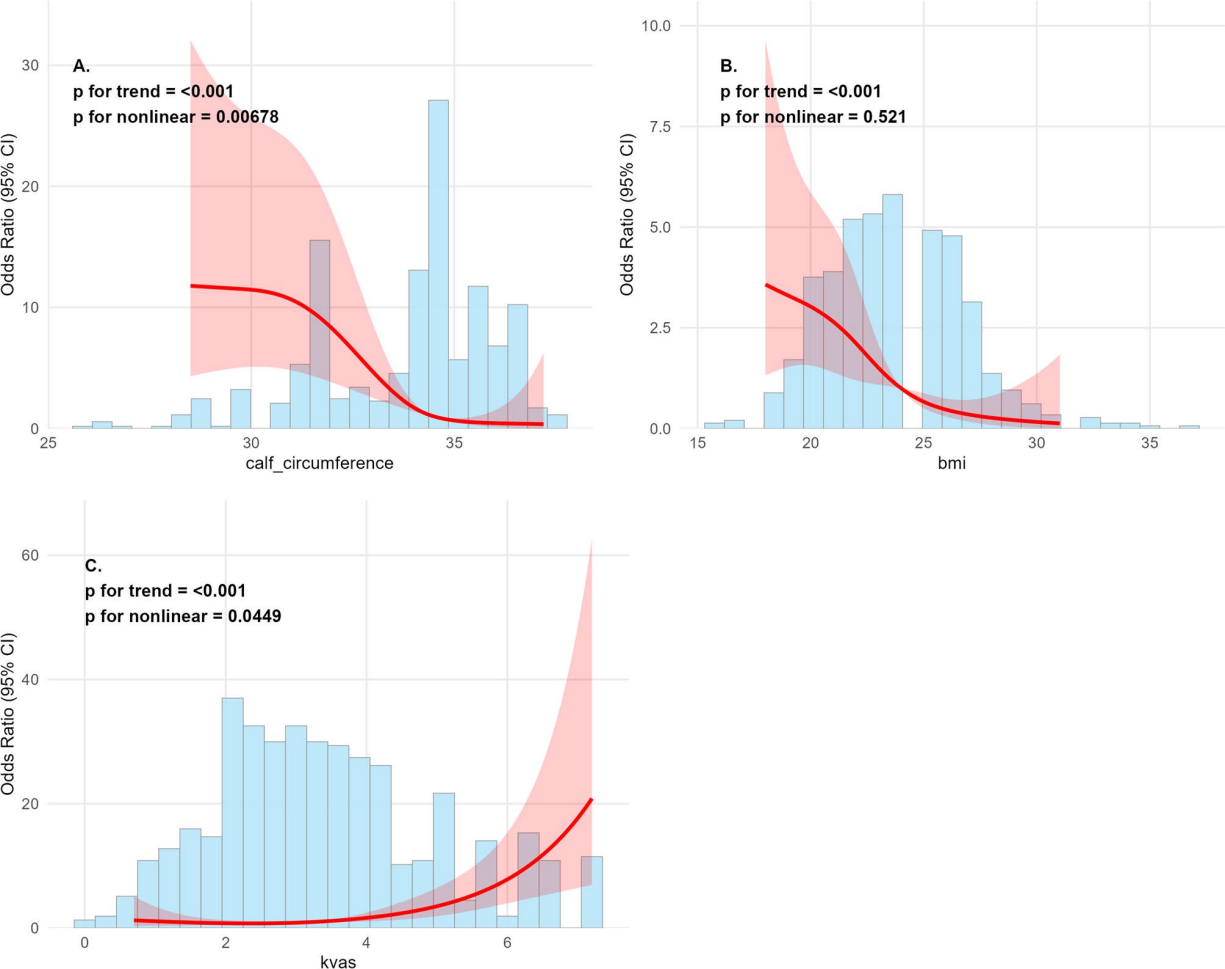
Nonlinear Associations of Continuous Predictors with SPKOA Risk. Panels **A**, **B**, and **C** show the nonlinear associations between calf circumference, BMI, and KVAS score, respectively, and the risk of sarcopenic knee osteoarthritis (SPKOA). The solid line represents the estimated odds ratios (ORs), and the shaded area indicates the 95% confidence intervals (CIs). All three variables exhibit significant nonlinear trends

Continuous predictors (CC, BMI, KVAS) showed significant nonlinear associations with SPKOA risk (Fig. 6): calf circumference: Risk increased sharply when < 32 cm; BMI: Risk peaked at 28–30 kg/m², then declined; KVAS: Risk rose exponentially with scores > 4.

### BPDKC model nomogram and calculator

**Fig. 7:**
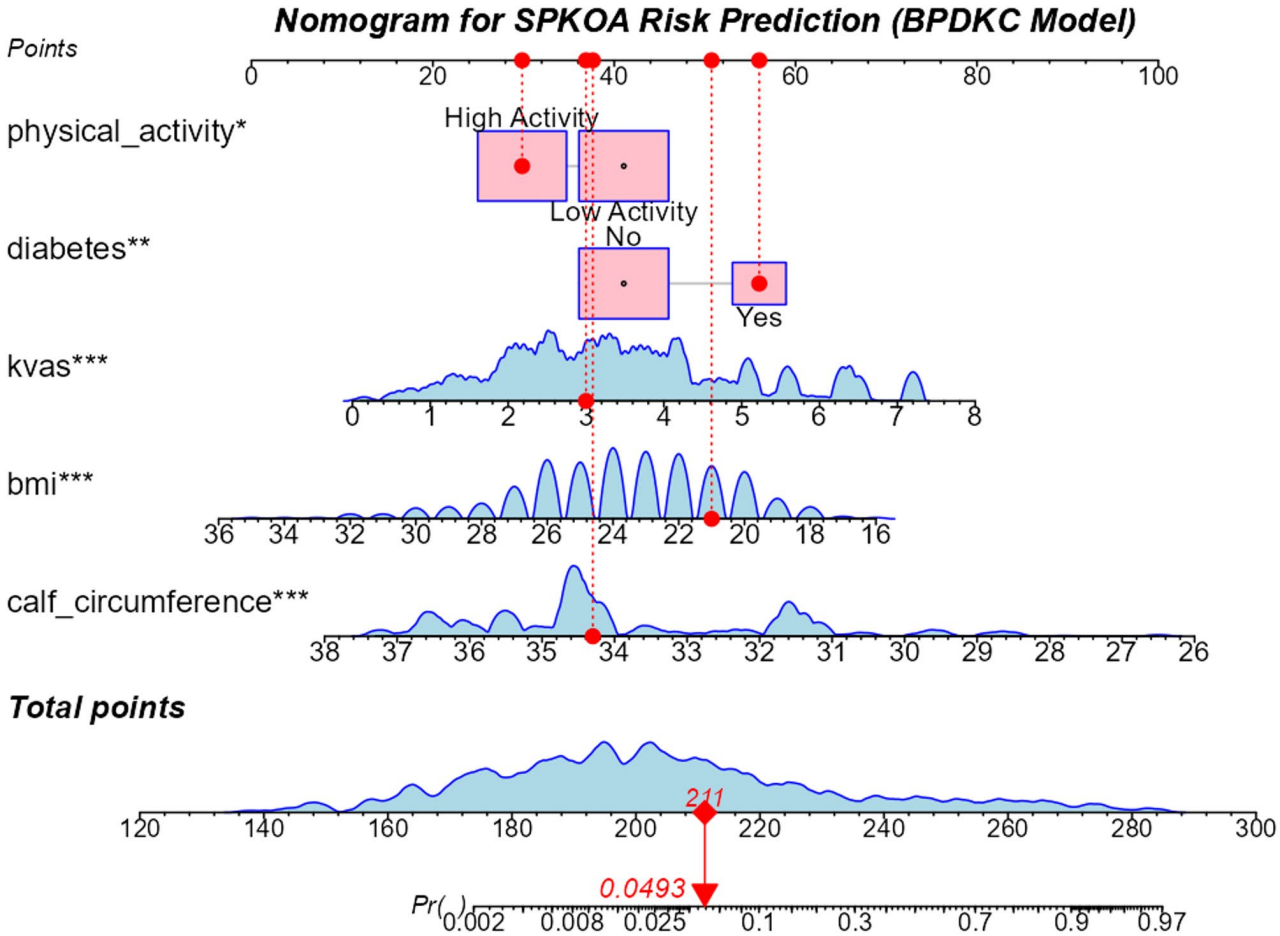
Nomogram for SPKOA Risk Prediction (BPDKC Model). The bottom scale shows the predicted probability of SPKOA (range: 0–1). To express as percentage, multiply by 100 (0.0493 = 4.93%)

A nomogram was developed to visualize Model 2, integrating predictors with assigned point ranges: BMI (0–30 points), physical activity (0–30 points), DM (0–40 points), KVAS (0–25 points), and calf circumference (CC, 0–100 points) (Fig. 7). Total points from all predictors map directly to the probability of SPKOA. For example, a total score of 160 corresponds to a 0.06% risk (probability = 0.0006), while a score of 211 corresponds to a 4.9% risk (probability = 0.049).

This tool enables clinicians to rapidly estimate individual SPKOA risk by integrating multimodal clinical and anthropometric data, facilitating personalized assessment.

**Fig. 8:**
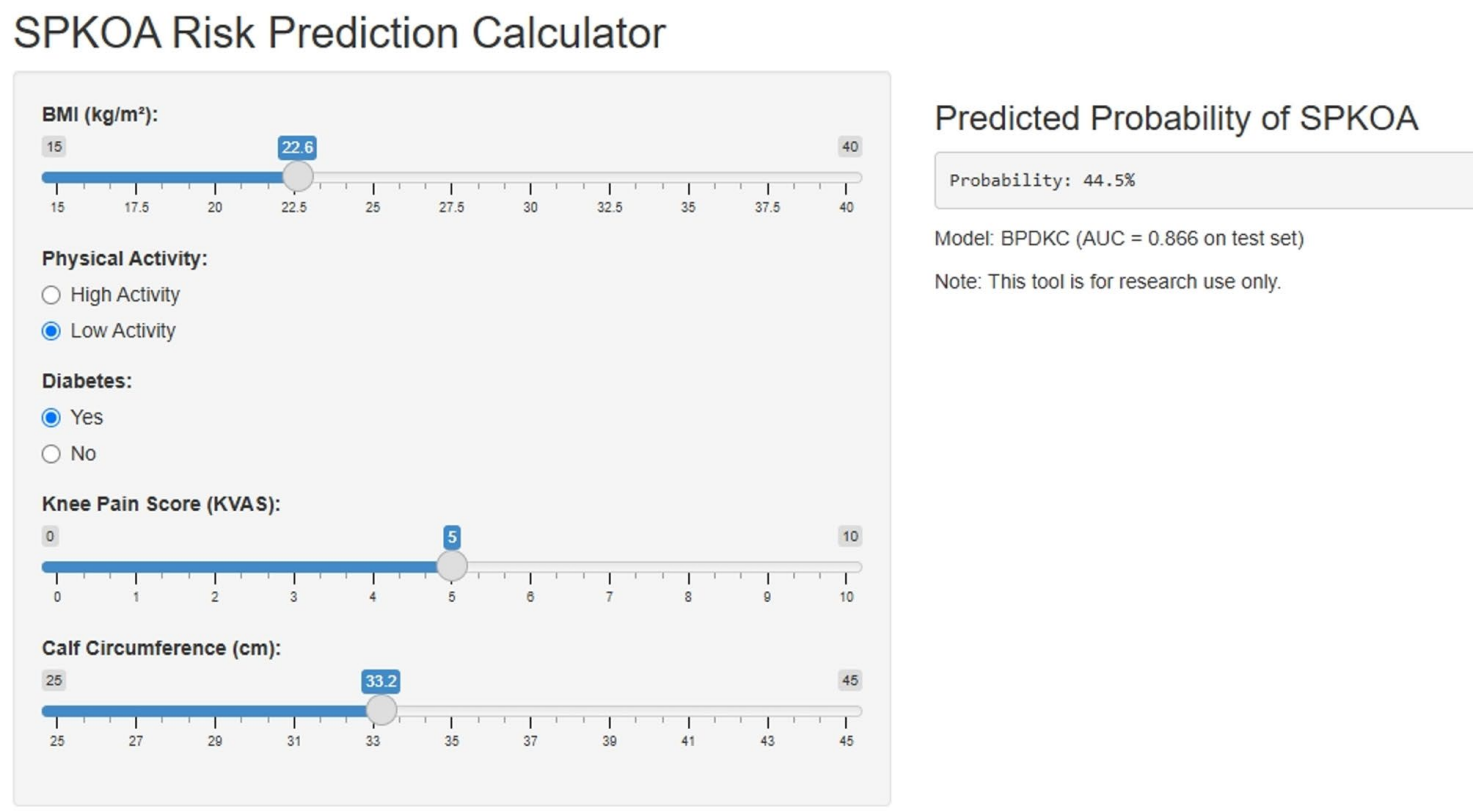
Web-based dynamic calculator. Note: This tool is for research use only, with the model achieving an AUC of 0.861 in the test set

An open-access web calculator (https://lgmyyyy.shinyapps.io/bpdkc_spkoa_app/*)* implements this nomogram, enabling real-time risk estimation. Note: The model’s AUC in the test set is 0.861; predicted probabilities > 40% are plausible only in high-risk profiles (Fig. 8).

## Discussion

### Epidemiological significance of SPKOA and current research gaps

This study reported a 12.5% prevalence of SPKOA in a community-dwelling older adult population, which is higher than the 8.6% reported in a previous study by Iijima and Aoyama [32, 33]. The discrepancy may be attributed to two key factors: first, differences in the age range of the study population, as SPKOA risk escalates with advanced age; second, more stringent screening methods in our research—integrating dual diagnostic criteria for SP and KOA to enhance detection sensitivity. This high prevalence underscores SPKOA’s role as a major contributor to disability in aging populations, further supported by our finding that patients with SPKOA had a 2.3-fold higher risk of falls compared to those with isolated SP or KOA.

Existing research has begun to recognize the interplay between muscle and joint health, though focus on SPKOA remains limited. Kristine Godziuk analyzed 11 studies [34], noting that muscle weakness, low skeletal muscle mass, and/or sarcopenia frequently co-oCCur with obesity in patients with osteoarthritis (OA), and suggested sarcopenic obesity should be considered in OA evaluation. Similarly, Nicola Veronese highlighted a significant association between SP and KOA, advocating for their recognition as distinct entities in future research and clinical practice [35]. Most SP-related studies have focused on developing clinical prediction models for isolated sarcopenia: Qianqian Gao [36] identified a comprehensive set of predictors, including sociodemographic variables (age, marital status, ADL disability, BMI), behavioral factors (smoking, PA levels, nutritional status), and disease-related factors (DM, cognitive impairment, osteoporosis); Shangjin Lin’s study [37] further identified CISD1, ETNPPL, and WISP2 proteins as potential SP biomarkers; Zongjie Wang [38] emphasized cognitive function and blood urea nitrogen (BUN) as critical predictors, alongside education level and chronic disease burden. These findings advance SP screening but leave a gap—few studies have addressed prediction models specifically for SPKOA. To our knowledge, this study is among the earliest in China to focus on SPKOA, using community-based surveys to identify predictive factors and develop a user-friendly nomogram and web calculator for risk assessment.

### Interpretation of BPDKC model predictors and unresolved questions

The BPDKC model, incorporating five predictors (BMI, PA, DM, KVAS, CC), addresses the need for SPKOA-specific risk assessment, though several observations and unresolved questions merit discussion.

BMI: exerted a significant effect on SPKOA, with low BMI (median 21 kg/m²) associated with a higher risk (*p* < 0.001). This contrasts with the well-established positive association between high BMI and isolated KOA—Huaqing Zheng [39] showed each 5 kg/m². BMI increase raises knee OA risk by 35%—while the relationship between low BMI and knee OA pain remains unclear. Molly Curtis [40] noted low BMI is linked to increased sarcopenia likelihood, which aligns with our finding that low BMI may contribute to SPKOA by exacerbating muscle loss. For example, BMI showed a significant group difference in Table 1 (median 21.0 vs. 24.0 kg/m², *p* < 0.001) and met inclusion criteria in univariate logistic regression; it was therefore retained in the multivariate model, where it remained independently associated with SPKOA (aOR 1.08 per unit decrease, *p* = 0.012). RCS modeling for BMI revealed a visually inverted U-shaped association peaking at 28–30 kg/m², though nonlinearity was not statistically significant (*p* = 0.521), supporting its linear retention in the model. This pattern — diverging from the typical obesity-driven KOA risk — may reflect the dual pathology of SPKOA, where low BMI signals sarcopenia-related vulnerability, and moderate adiposity may not confer the same joint-loading detriment seen in isolated knee OA.

**Table 1:** Baseline characteristics and univariate/multivariate logistic regression analyses for SPKOA in the total set

Characteristic	Overall	No SPKOA^a^	SPKOA^a^		Univariate		Multivariate	
	*N* = 640^a^	*n* = 560 ^a^	*n* = 80 ^a^	p-value^b^	OR (95% CI)^c^	p-value^d^	adjusted OR (95% CI)^c^	p-value^d^
Gender, n (%)				0.074				
Male	271 (42.34)	245 (43.75)	26 (32.50)		—		—	
Female	369 (57.66)	315 (56.25)	54 (67.50)		1.62 (0.99–2.69)	0.058	0.05 (0.01–0.20)	< 0.001***
Smoking, n (%)				0.193				
No	559 (87.34)	485 (86.61)	74 (92.50)		—		—	
Yes	81 (12.66)	75 (13.39)	6 (7.50)		0.52 (0.20–1.16)	0.144	0.64 (0.16–2.45)	0.513
Drinking, n (%)				0.202				
No	574 (89.69)	506 (90.36)	68 (85.00)		—		—	
Yes	66 (10.31)	54 (9.64)	12 (15.00)		1.65 (0.81–3.15)	0.144	1.98 (0.53–7.22)	0.298
Physical Activity, n (%)				< 0.001***				
Low Activity	333 (52.03)	277 (49.46)	56 (70.00)		—		—	
High Activity	307 (47.97)	283 (50.54)	24 (30.00)		0.42 (0.25–0.69)	< 0.001***	0.35 (0.15–0.80)	0.015*
Diet, n (%)				0.455				
Abnormal Diet	331 (51.72)	286 (51.07)	45 (56.25)		—		—	
Normal Diet	309 (48.28)	274 (48.93)	35 (43.75)		0.81 (0.50–1.30)	0.386	1.26 (0.56–2.85)	0.578
Hypertension, n (%)				0.221				
No	323 (50.47)	277 (49.46)	46 (57.50)		—		—	
Yes	317 (49.53)	283 (50.54)	34 (42.50)		0.72 (0.45–1.16)	0.18	1.64 (0.71–3.88)	0.247
DM, n (%)				< 0.001***				
No	458 (71.56)	419 (74.82)	39 (48.75)		—		—	
Yes	182 (28.44)	141 (25.18)	41 (51.25)		3.12 (1.94–5.05)	< 0.001***	4.84 (2.15–11.5)	< 0.001***
Coronary Heart Disease, n (%)				0.342				
No	472 (73.75)	417 (74.46)	55 (68.75)		—		—	
Yes	168 (26.25)	143 (25.54)	25 (31.25)		1.33 (0.79–2.18)	0.278	0.37 (0.13–0.95)	0.045*
Osteoporosis, n (%)				0.062				
No	438 (68.44)	391 (69.82)	47 (58.75)		—		—	
Yes	202 (31.56)	169 (30.18)	33 (41.25)		1.62 (1.00–2.62)	0.048*	0.73 (0.30–1.71)	0.477
KL Grade, n (%)				0.818				
Grade 1–2	48 (7.50)	41 (7.32)	7 (8.75)		—		—	
Grade 3	310 (48.44)	270 (48.21)	40 (50.00)		0.87 (0.38–2.23)	0.749	1.12 (0.27–5.60)	0.885
Grade 4	282 (44.06)	249 (44.46)	33 (41.25)		0.78 (0.34–2.01)	0.573	1.30 (0.32–6.42)	0.732
Age (years)	71.0 (68.0–74.0)	70.0 (68.0–74.0)	72.0 (70.0–75.5)	< 0.001***	1.10 (1.05–1.16)	< 0.001	1.10 (1.01–1.21)	0.037*
BMI (kg/m^b^)	24.0 (22.0–26.0)	24.0 (22.0–26.0)	21.0 (20.0–23.0)	< 0.001***	0.73 (0.66–0.80)	< 0.001	0.99 (0.82–1.19)	0.9
KVAS	3.3 (2.3–4.3)	3.1 (2.2–4.1)	5.1 (3.2–6.3)	< 0.001***	1.82 (1.56–2.14)	< 0.001	1.87 (1.49–2.41)	< 0.001***
Grip Strength (kg)	20.7 (16.6–27.4)	21.2 (17.3–28.2)	17.5 (15.3–22.4)	< 0.001***	0.92 (0.88–0.95)	< 0.001	1.03 (0.95–1.11)	0.496
Paces per minute	1.0 (1.0–1.3)	1.2 (1.0–1.3)	1.0 (0.8–1.2)	< 0.001***	0.01 (0.00–0.04)	< 0.001	0.00 (0.00–0.04)	< 0.001***
ASMI (kg/m^b^)	6.6 (5.9–7.4)	6.7 (6.1–7.5)	5.5 (5.4–5.9)	< 0.001***	0.19 (0.12–0.27)	< 0.001	0.04 (0.01–0.10)	< 0.001***
Calf Circumference (cm)	34.5 (31.8–35.5)	34.5 (33.2–35.5)	31.5 (30.8–32.5)	< 0.001***	0.62 (0.55–0.70)	< 0.001	0.64 (0.53–0.76)	< 0.001***

Categorical variables are presented as n (%). Continuous variables are presented as median (first quartile–third quartile; Q1–Q3), consistent with the revised format in Sect. 2.3.5 and 2.4.4

*KL* Kellgren-lawrence, *KVAS* Knee visual analog scale, *ASMI* Appendicular skeletal muscle index, *OR* Odds ratio, *CI* Confidence interval

^a^N values represent total, No SPKOA, and SPKOA groups, respectively

^b^Group comparisons: Wilcoxon rank-sum test for continuous variables, chi-square test for categorical variables

^c^Odds Ratio (OR) and 95% Confidence Interval (CI)

^d^Significance based on Wald test in logistic regression; *p < 0.05, **p < 0.01, ***p < 0.001

PA Level: High PA levels were beneficial for reducing SPKOA prevalence. In end-stage KOA populations, low activity and high BMI are major causes of muscle loss (as inactivity worsens atrophy and KOA symptoms), but our data suggest proper activity increases muscle strength without aggravating joint degeneration, thereby lowering SPKOA risk. SPKOA patients in our study reported significantly less exercise than non-SPKOA individuals, and targeted interventions (Tai Chi) were shown to reduce SPKOA occurrence—findings validated by the model.

DM: emerged as an important SPKOA predictor. A. Eitner [41] showed DM aCCelerates OA progression and increases knee pain severity; Seow, S [42] noted knee OA combined with DM reduces muscle strength and physical activity. Nearly all studies confirm a strong link between DM and sarcopenia: Liyuan Feng’s latest network meta-analysis [43] showed higher SP prevalence in diabetic patients, with risk factors including age, HbA1c, and diabetic nephropathy; Wouter Tack [44] highlighted shared pathophysiological mechanisms (muscle structure changes, chronic low-grade inflammation) between sarcopenia and type 2 DM.

KVAS: was a strong SPKOA predictor, with high test-retest reliability in knee arthritis pain measurement [45]. While L. Vlietstra [46] found VAS (used for fatigue assessment) had no significant association with sarcopenia muscle mass measures (limb muscle index) in OA/RA patients, Notably, KVAS emerged as one of the strongest and most consistent predictors of SPKOA in both univariate (OR = 1.82, *p* < 0.001) and multivariate analyses (adjusted OR = 1.87, 95% CI: 1.49–2.41, *p* < 0.001). Individuals with SPKOA reported significantly higher pain levels (median KVAS = 5.1, Q1–Q3: 3.2–6.3) compared to those without SPKOA (median = 3.1, Q1–Q3: 2.2–4.1), underscoring pain not merely as a symptom, but as a potential driver of the SPKOA phenotype. We hypothesize that severe knee pain leads to activity avoidance, which in turn accelerates muscle disuse, atrophy, and functional decline — thereby reinforcing the vicious cycle. This supports targeting pain management and early mobilization as key strategies in SPKOA prevention. RCS modeling identified a significant nonlinear pattern (*p* = 0.045), with risk rising exponentially beyond KVAS = 4 — suggesting not only a symptom marker but a quantifiable inflection point for early intervention in SPKOA, consistent with its strong directional association (p for trend < 0.001).

CC was a key predictor, consistent with its inclusion in clinical sarcopenia diagnostic criteria. Studies show SARC-Calf (incorporating CC) has higher specificity than SARC-F for sarcopenia risk identification [47]; Sunyoung Kim [48] suggested a 32 cm cutoff for CC as a good sarcopenia indicator in Korean elderly; Chung-Yao Chen [49] reported CC-based sarcopenia prediction with an AUC of 0.819. While few studies explore CC and knee OA directly, our data showed a clear association with SPKOA: the overall median CC was 34.5 cm (Q1-Q3: 31.8–35.5 cm), with non-SPKOA individuals having a median of 34.6 cm (Q1-Q3: 33.2–35.5 cm) vs. 31.5 cm (Q1-Q3: 30.8–32.5 cm) in SPKOA patients, indicating lower CC correlates with higher SPKOA prevalence. RCS modeling confirmed a statistically significant nonlinear association (*p* = 0.007), with risk sharply increasing below ~ 32 cm — consistent with its role in capturing sarcopenia — a core component of SPKOA — and supporting its utility as a practical, anatomy-based indicator for SPKOA risk stratification, alongside a strong directional trend (p for trend < 0.001).

### Unresolved questions for future research

Several factors known to associate with sarcopenia or knee OA did not retain significance in the final BPDKC model, warranting deeper exploration. Smoking, previously linked to stronger SP-OA associations in Peng et al. [12], showed no significant difference in our cohort — potentially reflecting regional behavioral patterns, survivor bias, or residual confounding by unmeasured variables such as pack-years or cessation timing. Similarly, KL grade — though a standard radiographic marker of KOA severity — was not predictive of SPKOA (overall *p* = 0.818), possibly due to its categorical coarseness or nonlinear relationship with functional decline; future studies should consider continuous imaging biomarkers or subgroup stratification to better capture structural-clinical discordance. Age, while significant in univariate analysis and selected in top BIC models, was excluded from the final model — likely because our narrow age range (65–85 years) restricted variability, masking its true effect in broader populations. Finally, despite a 3:1 female predominance in SPKOA prevalence (73% vs. 27%), multivariate modeling paradoxically suggested higher male risk — an artifact likely driven by model intercept and sample imbalance. This underscores the need for gender-stratified analyses in larger, balanced cohorts to clarify sex-specific pathophysiology and risk trajectories in SPKOA.

### Strengths of the BPDKC model

The BPDKC model demonstrates strong discriminative performance (AUC = 0.835 in the independent test set) for identifying older adults at high risk of SPKOA. Its core predictors — CC (adjusted OR = 0.64), KVAS (OR = 1.87), and DM (OR = 4.84) — reflect key pathophysiological pathways: CC serves as a simple, non-invasive proxy for lower-limb muscle reserve without diagnostic circularity; KVAS captures the “pain-inactivity-muscle loss” vicious cycle central to SPKOA progression; and DM highlights the role of metabolic dysregulation in accelerating both joint degeneration and muscle wasting. Requiring only five routinely assessable variables — BMI, CC, DM, KVAS, and PA — the model is highly feasible for real-world implementation in primary care or community settings, with no need for imaging or specialized equipment. The accompanying web-based calculator enables instant, user-friendly risk estimation, facilitating early, targeted interventions such as exercise promotion, nutritional support, or pain management before irreversible functional decline occurs.

### Mechanistic insights

DM emerged as a potent metabolic driver of SPKOA (adjusted OR = 4.84), likely through multiple synergistic pathways: chronic hyperglycemia promotes accumulation of advanced glycation end products (AGEs) in both muscle and cartilage, inducing fibrosis and impairing contractile and load-bearing function; concomitant insulin resistance disrupts anabolic signaling, accelerating muscle catabolism and further destabilizing the knee joint. This positions DM not merely as a comorbidity but as a modifiable upstream regulator of the sarcopenia-OA axis.

The non-significant association between BMI and SPKOA (adjusted OR = 0.99) reveals a pathophysiological divergence from isolated KOA — where obesity-driven mechanical stress dominates. In SPKOA, low BMI (median 21 kg/m²) often reflects malnutrition or cachexia, which directly fuels sarcopenia and overrides any protective mechanical buffering from higher adiposity. While extreme obesity may still contribute to joint wear, its effect is attenuated in SPKOA due to the loss of muscle-mediated joint stabilization. This aligns with emerging evidence that body composition metrics — such as fat-free mass index (FFMI) or fat mass-to-fat-free mass ratio (FM/FFM) — may better capture functional risk than BMI alone [50], suggesting future SPKOA models could integrate these for improved precision.

CC, the model’s strongest predictor (OR = 0.64), serves as a practical surrogate for lower-limb muscle mass — particularly quadriceps volume — and thus joint stability. Reduced CC directly links sarcopenia to accelerated knee degeneration: weaker muscles fail to absorb impact, increasing cartilage stress and pain, which further reduces activity and deepens muscle loss. Validated CC cutoffs (35 cm men, 33 cm women) for sarcopenia screening correlate with higher mortality risk [51], reinforcing its dual role as both a diagnostic and prognostic biomarker in SPKOA.

### Study limitations

Despite high specificity (0.946), the BPDKC model demonstrates critically low sensitivity (0.375) in the independent test set, indicating it fails to identify over 60% of true SPKOA cases — a major limitation for any screening tool where early case detection is essential. This poor sensitivity likely reflects both the small number of SPKOA cases (*n* = 80) and the model’s derivation from a single, homogeneous urban cohort in Shanghai, which — combined with strict inclusion criteria — artificially constrained clinical variability and inflated performance metrics. Crucially, the model lacks external validation; its AUC of 0.861 and calibration remain untested in demographically, geographically, or clinically diverse populations. Without multi-center or prospective validation, its generalizability — and thus real-world clinical utility — cannot be assumed. Future work must prioritize external validation, recalibration to improve sensitivity (via risk threshold adjustment or inclusion of inflammatory/psychological markers), and evaluation in primary care settings to determine whether the model can reliably support early intervention before irreversible functional decline occurs (Table 2).

**Table 2:** Performance metrics of the BPDKC model and four alternative models

Metric	Model Performance on Training Set			Model Performance on Test Set		
	Model 1	Model 2	Model 3	Model 1	Model 2	Model 3
AUC (95% CI)	0.953 (0.930,0.975)	0.951 (0.930,0.973,)	0.934 (0.904,0.964)	0.835 (0.760,0.909)	0.861 (0.795,0.926)	0.806 (0.723,0.890)
Sensitivity (95% CI)	0.571 (0.432, 0.703)	0.571 (0.432, 0.703)	0.536 (0.397, 0.670)	0.417 (0.221, 0.634)	0.375 (0.188, 0.594)	0.417 (0.221, 0.634)
Specificity (95% CI)	0.974 (0.954, 0.988)	0.964 (0.941, 0.980)	0.964 (0.941, 0.980)	0.935 (0.886, 0.967)	0.946 (0.901, 0.975)	0.952 (0.908, 0.979)
Precision (95% CI)	0.762 (0.605, 0.879)	0.696 (0.542, 0.823)	0.682 (0.524, 0.814)	0.476 (0.257, 0.702)	0.500 (0.260, 0.740)	0.556 (0.308, 0.785)
Recall (95% CI)	0.571 (0.432, 0.703)	0.571 (0.432, 0.703)	0.536 (0.397, 0.670)	0.417 (0.221, 0.634)	0.375 (0.188, 0.594)	0.417 (0.221, 0.634)
F1 Score (95% CI)	0.653 (0.537, 0.767)	0.627 (0.506, 0.725)	0.600 (0.467, 0.708)	0.444 (0.259, 0.627)	0.429 (0.243, 0.618)	0.476 (0.294, 0.667)
Brier	0.0532 (0.038, 0.068)	0.0582 (0.043, 0.074)	0.0620 (0.046, 0.078)	0.1064 (0.069, 0.143)	0.1038 (0.068, 0.139)	0.1027 (0.067, 0.138)
AIC	172.55	178.41	194.79			
BIC	205.39	203.04	219.42			

Note: Confidence intervals (CIs) for AUC, Sensitivity, Specificity, Precision, Recall, and Brier Score were calculated using the exact binomial test (Clopper-Pearson method) for proportions or the normal approximation for Brier Score. The 95% CI for the F1 score was estimated using the bias-corrected and aCCelerated (BCa) bootstrap method with 500 resamples. AIC and BIC are reported without CIs as they are point information criteria

Model 1 variables: CC, KVAS, BMI, DM, PA, osteoporosis, age

Model 2 variables: BMI, PA, DM, KVAS, CC

Model 3 variables: DM, PA, KVAS, CC, age

## Supplementary Information

Supplementary Material 1.

Supplementary Material 2.

Supplementary Material 3.

Supplementary Material 4.

Supplementary Material 5.

## Data Availability

The data that support the findings of this study are available from the corresponding author upon reasonable request.
