# Supplementary material for: Development and validation of the BPDKC model for predicting sarcopenic knee osteoarthritis in community-dwelling older adults: a cross-sectional study
Source: BMC Musculoskelet Disord. 2025 Nov 26;26:1072. doi: 10.1186/s12891-025-09311-6 (PMC12659516; doi:10.1186/s12891-025-09311-6)
Supplement: Supplementary file 1 — Supplementary Material 1. [file 12891_2025_9311_MOESM1_ESM.pdf]

AF-SOP19-F-1.0

研究项目伦理审查批件

TRIAL ETHICS COMMITTEE APPROVAL FORM

XSEC2023010

研究项目名称<br>PROTOCOL TITLE	老年人肌少性膝关节炎多因素临床预测模型的开发和验证		
项目负责人<br>PRINCIPAL INVESTIGATOR	刘光明	科室<br>DEPARTMENT	骨伤科
研究时间<br>DURATION OF THE STUDY	2023.12.1-2026.11.30		
项目资助及编号<br>SPONSORED BY<br>PROJECT/NO.	2023-2026 年度黄浦区卫生健康系统专业人才梯队建设项目培养对象——骨干人才（2023BJ05）		
项目承担单位（协作）<br>INSTITUTE	上海市黄浦区豫园社区卫生服务中心、上海市黄浦区豫园街道社区卫生服务中心、上海中医药大学附属曙光医院		
审查形式<br>WAY OF REVIEW	☑会议审查 Board Meeting　☐快速审查 Expedited Review		

投票结果 （快速审查不填写此项）

VOTING (Expedited Review do not fill this)

本次会议应到人数：11 人，实到人数：11 人，参加投票人数：11 人，回避：0 人。

同意：7 票，修改后同意：4 票，不同意： 0 票，终止或者暂停已同意的研究：0 票。

委员会审批意见

COMMENTS OF ETHICS COMMITTEE

[√] 批准 Approval
[ ] 修改后批准 Approved with Recommendation
[ ] 修改后再审 Modified retrial
[ ] 不批准 Rejected
[ ] 终止或者暂停已同意的研究 Termination or suspension of approved research

跟踪审查频率 Frequency of continuing review:

（1）三个月 3 months ☐（2）六个月 6 months ☐（3）十二个月 12 months ☑
（4）其他 others ______________☐

主任委员签名 Signature of Chairperson:

伦理委员会（盖章）Seal of Ethics Committee:

审批日期 Date: 2023 年 12 月 28 日

联系地址：上海市复兴中路 528 号
Address: NO.528 Middle Fu Xing Road, Shanghai
电话/传真：53061730
Tel/Fax: 53061730

研究项目伦理审查批件

TRIAL ETHICS COMMITTEE APPROVAL FORM

XSEC2023010

审查材料 The following items have been reviewed

1. 研究者的资格、经验是否符合试验要求；
2. 研究方案是否符合科学性和伦理原则的要求；
3. 受试者可能遭受的风险程度与研究预期的收益相比是否合适；
4. 在办理知情同意过程中，向受试者（或其家属、监护人、法定代理人）提供的有关信息资料是否完整易懂，获得知情同意的方式是否恰当；
5. 对受试者的资料是否采取了保密措施；
6. 受试者入选和排除的标准是否合适和公平；
7. 是否向受试者明确告知他们应享有的权益，包括在研究过程中可以随时退出而无须提出理由且不受歧视的权利；
8. 受试者是否因参加研究而获得合理补偿，如因参加研究而受到损害甚至死亡时，给予的治疗以及赔偿措施是否合适；
9. 研究人员中是否有专人负责处理知情同意和受试者安全的问题；
10. 对受试者在研究中可能承受的风险是否采取了保护措施；
11. 研究人员与受试者之前有无利益冲突。

审查委员名单 Member List

姓名	所在单位	任职	职称
柴丽萍	上海市黄浦区香山中医医院	主任委员	主治医师
张文婷	上海市黄浦区香山中医医院	副主任委员	主治医师
刘炎	上海市黄浦区香山中医医院	副主任委员	副主任医师
李辰	上海市黄浦区香山中医医院	委员	主治医师
石燕	上海市黄浦区香山中医医院	委员	主管护师
刘嫦溥	上海市黄浦区香山中医医院	委员	副主任医师
孙波	上海市黄浦区香山中医医院	委员	主任医师
杨骏	上海市黄浦区香山中医医院	委员	主任药师
沈德海	上海市黄浦区香山中医医院	委员	副主任医师
张梦圆	上海市黄浦区香山中医医院	委员	主治医师
罗宏华	上海市黄浦区香山中医医院	委员	主管护师

联系地址：上海市复兴中路 528 号
Address: NO.528 Middle Fu Xing Road, Shanghai
电话/传真：53061730
Tel/Fax: 53061730

研究项目伦理审查批件

TRIAL ETHICS COMMITTEE APPROVAL FORM

XSEC2023010

声明 Statement:

1、本伦理委员会的相关工作遵循中华人民共和国食品药品监督管理局颁发的药物临床质量管理规范(GCP)和 ICH-GCP 的伦理审查原则，符合赫尔辛基宣言的要求，并遵守中国相关法律和法规的的规定。
2、审查结果为非"同意"，申办者和研究者可在 1 个月内作相关修改，并陈述理由。
3、研究过程中，对方案和知情同意书等相关文件的任何修改，需经伦理委员会审查同意后方可实施。
4、本中心发生的严重不良事件或非预期不良事件需向本伦理委员会通报，并等待做出新的决定。
5、自批准函生效日起，批件有效期为 1 年，在有效期内未启动的项目需要重新进行审查，需要请研究者在规定的持续审查日期到期前 1 个月递交进展报告，以获得批准持续进行研究。研究结束后，需递交总结报告。
1、Xiangshan TCM Hospital Ethics Committee conducts ethical review according to the Chinese GCP, ICG-GCP, Declaration of Helsinki and other local and international guidelines.
2、If the decision is not "Approval", PI should reply within 1 month or clarify the reasons.
3、Any changes in the documents should be approved before implement.
4、Any SAE or unexpected AE happening in this site should be reported to the EC. Decision will be followed.
5、This protocol is approved for 1 year since the date. If the study doesn't start with 1 year, it should be reviewed again. Please submit the progress report 1 month before the deadline. Please submit the closing report when study is over.

联系地址：上海市复兴中路 528 号
Address: NO.528 Middle Fu Xing Road, Shanghai
电话/传真：53061730
Tel/Fax: 53061730
